# Seeing a sunset: Exploring the joy of vision, in healthy eyes and ocular disease

**DOI:** 10.1111/opo.70019

**Published:** 2025-09-16

**Authors:** Andrew J. Anderson, Lauren N. Ayton, Eden G. Robertson, Bao N. Nguyen

**Affiliations:** 1 Department of Optometry and Vision Sciences, The University of Melbourne, Parkville, Victoria Australia; 2 Department of Surgery (Ophthalmology), The University of Melbourne, Parkville, Victoria Australia; 3 Centre for Eye Research Australia, Royal Victorian Eye and Ear Hospital, East Melbourne, Victoria Australia; 4 Discipline of Paediatrics and Child Health, School of Clinical Medicine, Faculty of Medicine and Health, University of New South Wales, Kensington, Western Australia Australia; 5 Children's Medical Research Institute, Westmead, New South Wales Australia

**Keywords:** ocular disease, qualitative, vision loss, visual enjoyment, visual function, wellbeing

## Abstract

**Purpose:**

Vision plays a critical role in the performance of various functional tasks, and can also be an inherent source of enjoyment unrelated to a functional task. This study aimed to explore the sources and importance of visual enjoyment and how these might alter with vision loss.

**Methods:**

Fourteen adults (26–81 years) with self-reported healthy vision and 15 (37–84 years) with vision loss (inherited retinal disease, glaucoma, age-related macular degeneration) participated. Across four focus groups (2 × healthy vision, 2 × vision loss), participants were asked about sources of visual enjoyment, attitudes around the distinction between vision to perform tasks versus vision as an inherent source of enjoyment, how sources of visual enjoyment may have changed through eye disease or aging, and experiences with eye care providers regarding visual enjoyment. Transcriptions were analysed using qualitative content analysis.

**Results:**

Almost all participants felt sources of visual enjoyment were important. Most could think of examples of visual enjoyment as distinct from visual function (e.g., appreciating leaf colour changes, stargazing), with a minority noting sources of enjoyment that either depended upon, or were facilitated by, good vision (e.g., playing golf, reading). Although around half believed the distinction between visual enjoyment and visual function was important, some were unclear whether there was a distinction or saw no distinction. Most felt that aging and vision loss with ocular disease had altered what they considered as sources of visual enjoyment. While direct experience of eye care practitioners considering personal sources of visual enjoyment when providing advice was mostly lacking, many respondents felt visual enjoyment would be important or beneficial to consider.

**Conclusions:**

Sources of visual enjoyment are important to most people with and without vision loss. These results suggest that some patients may value having their personal sources of visual enjoyment considered by eye care providers.

**Supplementary Information:**

The online version of this article (doi:10.1111/opo.70019) contains supplementary material, which is available to authorized users.

## Key points

- This study proposes a framework for distinguishing between enjoyment arising from the visual sense itself (inherent visual enjoyment) and enjoyment from activities facilitated by, or involving, vision (visually facilitated enjoyment).
- Most participants could identify situations where their visual sense itself gave them joy, rather than simply allowing them to perform enjoyable activities.
- Most participants reported that their eyecare provider did not consider their personal sources of visual enjoyment when providing advice, although many felt that adding this consideration would be valuable.

## BACKGROUND

Globally, 474 million people are predicted to have moderate to severe visual impairment by 2050, with a further 360 million predicted to have mild vision impairment.^1^ The impact of vision impairment on daily tasks can be assessed through functional tests.^2,3^ In addition, impact is commonly assessed through questionnaires, such as the US National Eye Institute Visual Functioning Questionnaire – 25 (VFQ-25),^4^ aimed at quantifying a person's quality of life (QoL). The increased use of QoL measures in the vision literature is—in part—due to regulating agencies, such as the US Food and Drug Administration, encouraging the use of patient-reported outcome measures.^5^

Most QoL assessments concentrate on the impact that vision loss has on various daily tasks for which vision is a factor (e.g., driving, navigating an environment, recognising people).^4,6,7^ As such, these assessments might be considered largely functional measures. Most assessments consider recreational activities, although typically these also involve performing functional tasks for which vision is involved. Task examples include key visual dimensions of reading (reading text, tape measures, maps and recipes for the leisure activities of reading books, sewing, sightseeing and recreational cooking), mobility (hiking, playing sport and sightseeing), visual information processing (identifying plants and weeds, seeing material grain in leatherwork and woodworking, seeing a subject or model when painting) and visual motor processing (knitting, baiting a hook for fishing, going skiing).^8^ Previous focus group work has also centred on the impact of vision loss on functional activities.^9^

However, vision has value beyond facilitating the completion of particular functional tasks, including recreational and leisure tasks, and can be itself a source of enjoyment. For example, over a quarter of Australians visit art galleries and museums each year, with this proportion being even higher within older age groups,^10^ suggesting that observing visual art is a common source of visual enjoyment largely separated from functional activities. While a distinction between vision purely for performing functional tasks (e.g., reading labels) and vision purely for enjoyment (e.g., viewing artwork) likely exists, the level of distinction for many tasks is unclear and subjective. However, in an extreme example, the role of functional vision and vision for enjoyment can be separated completely: in a study where most blind patients receiving retinal prostheses (‘bionic eyes’) later abandoned them due to a lack of functional utility, those who persisted commonly did so for the ‘contemplative vision’ it provided for tasks that were of no functional consequence.^11^

While the impact of vision loss on functional tasks has been well explored,^12^ how vision loss might influence sources of enjoyment—particularly those derived directly from the visual sense, such as appreciating a sunset—has been comparatively underexplored. Recent work has examined the impact of vision loss on engagement with visual art, albeit within a small group of participants.^13^ A broader exploration of visual enjoyment sources would be valuable, as it may inform the development of assessment tools that capture more fully all aspects of vision and, consequently, the impact of vision loss. Patient-reported outcome measures need to appropriately reflect the daily lived experience of people.^5^ Furthermore, having a better understanding of what sources of visual enjoyment are, and how important they are to people, might help remediate a perceived lack of empathy from medical professionals caring for people with a vision impairment.^9^

Here, structured focus groups were used to explore the following research questions:

1. What do people with healthy vision and ocular disease consider to be sources of visual enjoyment and how important are these sources to them?
2. How do sources of visual enjoyment change over time, due to eye disease or normal aging?
3. How do people with healthy vision and ocular disease distinguish between vision to perform tasks versus vision as an inherent source of joy, and what is the importance of this division?
4. What experiences and preferences do people with healthy vision and ocular disease have regarding the consideration of visual enjoyment by eye care providers?

## METHODS

The project received institutional ethics committee approval (The University of Melbourne Human Research Ethics Committee ID #28687) and was conducted in accordance with the Declaration of Helsinki. A qualitative descriptive study was used using structured focus groups to answer the research questions, and followed the Consolidated criteria for Reporting Qualitative research (CoREQ) checklist for reporting.^14^

### Participants

This study used purposive sampling to recruit adults (*≥*18 years of age) with self-reported healthy vision and those with a confirmed ocular disease diagnosis. People with self-reported healthy vision were recruited through advertisements at The University of Melbourne, the Melbourne Eyecare Clinic, an internal database of previous research participants who had consented to being contacted by email or phone for future studies, and by word-of-mouth. If required, people in the healthy vision group had a clinical examination with an optometrist at The University of Melbourne to confirm no ocular disease and normal vision.

Participants with a confirmed ocular disease diagnosis were recruited through the Centre for Eye Research Australia's Open Eyes database, who had consented to being contacted by email or phone by researchers for potential study participation. Participants were recruited from 30th April to 28th May 2024. A sample size of 12 participants with self-reported healthy vision (i.e., two focus groups of six participants) and 12 participants with ocular disease (i.e., two focus groups of six participants) was deemed appropriate to achieve information power (i.e., ‘enough’ data to tell a meaningful story), given the specific study aims and the quality of dialogue.^15^ All participants were required to be fluent in English and consent to participation.

### Demographic and clinical information

Basic demographic data (visual acuity and an ocular disease diagnosis where relevant) were obtained through the participant's latest healthcare records with their consent, with age and sex collected by self-report. Participants completed the revised 28-item Impact of Vision Impairment Profile (IVI) questionnaire^16^ as a validated scale for measuring perceived restriction of participation associated with activities of daily living.

### Structured group discussions

Participants were allocated to one of four focus groups. Two focus groups consisted of only people with self-reported healthy vision, while the other two focus groups comprised individuals with a confirmed ocular disease diagnosis. This choice was principally due to the two participant types receiving different versions of Question 4, detailed below. Focus groups were conducted in-person at the Centre for Eye Research (CERA) in Melbourne. In attendance were the study participants, the facilitators (AJA, BNN) and an observer (LA), with a small group of students and staff on hand to provide general assistance (e.g., guiding participants within the building). At the start of each session, the lead author (AJA) gave a verbal pre-briefing (see Appendix S1) to ensure that all participants understood the study format, objectives, the terms that would be used in the focus group discussion (‘inherent source of enjoyment’, ‘ability to perform functional tasks’) and the goals, interests and reasons for conducting the research. This briefing also reminded participants of the voluntary nature of their involvement, and that their comments would be de-identified.

For their respective focus groups, the facilitators (AJA, BNN) followed a structured guide and set of questions to direct the group discussion (see Appendix S2). AJA is a male researcher and optometrist (BSc(Optom), MSc(Optom), PhD), with experience and research interest in visual psychophysics and visual aesthetics. BNN is a female researcher and optometrist (BOptom, PhD), with experience and research interest in the effects of aging and disease on the human visual system. There were no prior relationships between the participants and facilitators prior to the focus group discussions. Each participant was prompted at least once to provide a response for each of the five primary topics presented in the following order: (1) initial thoughts regarding an activity or situation associated with visual enjoyment; (2) importance of the distinction between vision for enjoyment and vision for function; (3) current sources of visual enjoyment and importance thereof; (4) change in sources of visual enjoyment with aging (for the participants with healthy vision) or vision loss (for the participants with an ocular disease diagnosis) and (5) eye care providers' consideration of visual enjoyment. Questions were not repeated, but the facilitator requested participants to clarify their responses where necessary and within time limitations (see Study Limitations). Discussions were divided into two (before and after a lunch break), with each part taking no more than 45 min.

Field notes were not made during the focus group sessions. However, after a debriefing session at the end of the focus group, participants were invited to provide written responses to three written questions (‘Are there additional sources of visual enjoyment for you that were not discussed in the workshop?’, ‘If so, can you briefly describe how these have or have not been impacted by any vision loss you may have?’ and ‘Are there any additional things you would like to share that you think it is important for us to know as we consider sources of visual enjoyment and how these might be influenced by vision loss?’). Assistance with these written documents was provided, as needed. Responses were included in the qualitative analysis.

### Data analysis

Demographic data were summarised using descriptive statistics and compared between groups using GraphPad Prism (version 10.3.1, graphpad.com). The focus group discussions were recorded with participant consent using Zoom (version 5.15, zoom.com) and then transcribed verbatim (BNN, AJA). Transcripts were not returned to the participants for correction or comment, given the group nature of the study and that the ethics approval did not permit sharing of recordings, which would be necessary to verify verbatim transcriptions. Following Elo and Kyngas' approach to a deductive qualitative content analysis,^17^ three authors (AA, BNN, LA) reviewed the transcriptions multiple times to become familiar with the data. Through discussion, an unconstrained categorisation matrix was developed as a framework to code the data (Table S1). To minimise bias in reporting, a second author without clinical experience or connection to the participants (EGR) then coded each transcription line-by-line using NVivo (version 14, lumivero.com). EGR iteratively revised the matrix categories through the process of grouping, categorising and abstraction. BNN then independently coded the transcriptions using the revised matrix (*k* = 0.65, 95% CI [0.61–0.69]), indicating a substantial level of agreement. Coding scores with a negative kappa, indicating poor agreement (<5% of cases), were reviewed by EGR and BNN and revised through discussion. Through discussion with the wider authorship team, a final code was added (reported in the section ‘A positive perspective on vision loss’). Participants did not provide feedback on the findings.

Despite content analyses typically allowing researchers to count the frequency of codes, the nature of the focus groups made it challenging to determine whether a response was missing as part of data collection (e.g., a participant did not specifically address the question) or truly missing from a participant's experience.^18^ To minimise any misrepresentation of the data, quantitative descriptors were used rather than exact counts to provide an indicator of salience and sense of the general patterns in the data.^19,20^ Where possible, exact frequencies were reported and matrix coding was used to explore any patterns between the two groups (self-reported healthy vision vs. confirmed ocular disease diagnosis).

**FIGURE 1:**
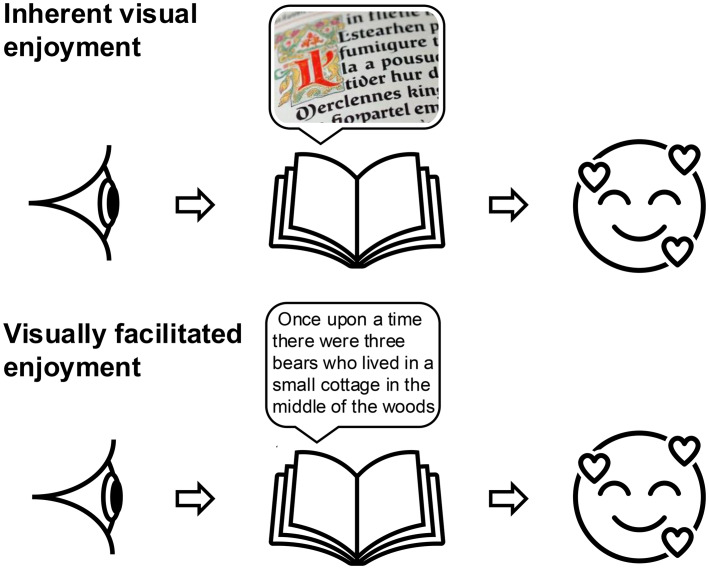
Examples demonstrating the ideas of inherent visual enjoyment (upper panels) and visually facilitated enjoyment (lower panels) used in the current paper. While they share the basic task of examining text on a page, enjoyment comes directly from the visual sense in inherent visual enjoyment (admiring calligraphy in an ancient, illuminated manuscript). In visually facilitated enjoyment, vision provides the means to perform the enjoyed activity (engaging with a story). While it is often possible to imagine performing a visually facilitated enjoyment task using non-visual means (for example, listening to an audiobook), this is typically not possible with an activity providing inherent visual enjoyment.

Throughout the analysis, an attempt was made to distinguish ‘visually facilitated enjoyment’ from ‘inherent visual enjoyment’ (Figure 1). ‘Visually facilitated enjoyment’ was defined as activity-derived enjoyment that is not visual per se, although the activity is typically done or facilitated by vision. Examples might include reading or playing golf. ‘Inherent visual enjoyment’ was defined as enjoyment that is derived from the visual sense itself (e.g., viewing a sunset or an artwork). Commonly, it would be possible to imagine enjoying a visually facilitated activity using a non-visual aid (for example, reading using audio books or having a sighted aid assist with localisation in golf), yet such substitution would likely not be successful for instances of inherent visual enjoyment.

## RESULTS

### Participants

Of the 18 persons with vision loss who were invited to participate, three declined. For those persons in the healthy vision group who were directly invited to participate (17), 11 declined due to unavailability on the day of the scheduled sessions. Persons with healthy vision also came from responses to public advertisement, for which a decline rate could not be determined. Participant demographic and clinical information appears in Table 1. Fifteen people with a confirmed ocular disease diagnosis (glaucoma, age-related macular degeneration and inherited retinal disease) were split into two focus groups of seven or eight participants, and 14 people with self-reported healthy vision were split into two focus groups of seven participants. The two groups did not differ in age (*p* = 0.17), with most participants being over 60 years of age (median age = 70 years). Around half of the participants were female (*n* = 15, 52%). The ocular disease group had worse visual acuity and perceived impact of vision impairment on their daily life activities (*p* < 0.01).

**TABLE 1:** Demographic and clinical information for the groups with ocular disease and self-reported healthy vision.

	Ocular disease group (*n* = 15)	Self-reported healthy vision group (*n* = 14)	*p*-Value
Age			
Years, median (range)	70 (37–84)	68 (26–81)	0.17^a^
Sex			
Female	8	7	
Male	7	7	
Other	0	0	0.86^b^
Main cause of vision loss		–	
Macular dystrophy	1		
Stargardts	2		
Cone-rod dystrophy	1		
Primary open angle glaucoma	4		
Neovascular AMD	2		
Geographic atrophy AMD	5		
Visual acuity			
Right eye, median (range)	6/12++ (6/240–6/4.8+)	6/6 (6/9.5–6/4.8+)	0.001^a^
Left eye, median (range)	6/7.5 = (6/240–6/4.8++)	6/6 (6/7.5–6/4.8)	0.006^a^
28-item IVI scale			
Score, median (range)	60 (10–86)	81 (55–84)	0.007^a^

*Note*: Lower scores on the Impact of Vision Impairment scale indicate a greater perceived negative impact of vision impairment on a person's quality of life.

Abbreviations: AMD, age-related macular degeneration; IVI, impact of vision impairment.

^a^Mann–Whitney rank sum test.

^b^Chi-square test of proportions.

### Initial perceptions around visual enjoyment

When invited to share an activity or situation associated with visual enjoyment, most participants provided examples that were considered in this analysis to be of ‘inherent visual enjoyment’. For example, sources of inherent visual enjoyment were commonly the joy in seeing the natural environment such as the ocean or forests, watching TV/movies or sport or seeing the faces of loved ones.

"For me it's nature… I don't know if any of you know [name of location]… it's really one of the most magnificent places. Just standing looking at the sunlight, the snow. Every time we go there, it's a completely different vista depending on the time of year. Now is the most beautiful time. All the trees, myrtle, all the leaves changing." 

65-year-old female with primary open angle glaucoma.

"I enjoy a lot… when I have a video call with my family back to my country… Seeing that picture on my phone or my computer makes me very happy." 

31-year-old male with healthy vision.

Around half of the participants across both groups also provided examples that were related more to ‘visually facilitated enjoyment’, with the activity requiring vision to serve a direct function (e.g., exercise, cooking) that led to enjoyment. Common examples of such visually facilitated enjoyment included the joy in playing sports, running and arts and craft.

"What I've enjoyed for a long time and can still do it, fortunately… I'm a long distance runner… What I'm finding now, where vision is helping me now… I can run 10K around the bush much easier because my vision, I can still see. And when I'm doing a long run, like 15, 20, 25K or something like that, covering a lot of distances… your vision helps you. I can see things like that. So I can still enjoy that." 

77-year-old male with geographic atrophy.

While most participants could provide at least one example of an activity or situation associated with visual enjoyment, two participants with healthy vision responded by discussing their vision for general functioning. One highlighted the importance of their vision to maintain independence and the other focussed on what they could no longer see due to their perception of their aging eyes.

"I find as you age, as I'm aging… [I] have friends with macular degeneration and other impairments… I just value… being able to live totally independently, not having to rely on anyone else for driving or any activities. It allows me just to live my life the way I want to be able to live it." 

72-year-old female with healthy vision.

"What I… find… really more frustrating [is] when I can't read labels… If I can read the street signs, if I can read a map, then I enjoy it. [But if I] have to put glasses on, I just find it at every turn, I'm just frustrated, frustrated, frustrated. Can't read it, got to put the glasses on… I never thought about, am I enjoying it? All I'm thinking about is what I can't see."

76-year-old female with healthy vision.

### Distinction between vision for enjoyment or function

Around half of the participants across both groups perceived a clear distinction between vision to perform particular tasks and vision as a source of enjoyment, and could conceptualise the difference by juxtaposing specific examples.

"For example… our family loves sport. And my children. I've got one cheerleader and one football player. So functionally, sometimes it's hard to get them around to the game or get myself to the game [because of my vision impairment]. But in the enjoyment factor, when I get to the game, can I actually see them play or see what they're doing? That's probably the enjoyment."

47-year-old female with Stargardt disease.

"I play golf. Two ways, you know. One, I'm out there thinking, talking to the girls: lovely day, isn't it? Gorgeous, aren't we lucky?… But the other thing is… I've actually got to find [the ball]. And so that's the other aspect of using my vision… trying to keep track of where the ball is. So quite different things that I'm visualising." 

76-year old female with healthy vision.

Several participants acknowledged a distinction in the two concepts but felt that they were so intertwined that it was difficult to separate one from the other.

"It does make me think about… how different it is, if there is any differential between use of vision as a tool for daily life or your use of vision to see something beautiful to enjoy. So from what we have discussed, looks like the two things are combined for me. I can see, OK, they are kind of, they're connected. Of course they're connected. You can't really simply separate them." 

44-year-old female with healthy vision.

While several participants commented on the importance of both vision to perform a task and vision as a source of enjoyment, several participants deemed ‘the functional aspect of vision’ as more important than inherent visual enjoyment. A few participants described a sequential nature, with function being a prerequisite for visual enjoyment to be possible.

"I value… vision more as a function. Enjoyment comes after… we satisfy our basic needs. So, I would value vision more as a function… to carry out our day-to-day activities and basic chores… Without your basic function, then… the enjoyment comes at the later stage."

26-year-old male with healthy vision.

Few participants perceived it was not important to distinguish between vision for function and vision for enjoyment. Specifically for participants with an ocular disease diagnosis, several commented that a distinction was irrelevant.

"I'm just one person, but…enjoyment versus functionality. It doesn't matter without vision. It's going to affect both."

37-year-old female with macular dystrophy.

"Maybe we're a bit fortunate to be able to sit here and think, well…is there a division? Is it the same? … The general population with good vision wouldn't even address that question. So, for me, there's no distinction… Everything is equally important. It kind of fits under the banner of everything rolled into one… there's no separation. It's in one box."

80-year-old female with primary open angle glaucoma.

There were no clear patterns between how the participant groups distinguished (or did not distinguish) the two aspects of vision. However, a few participants with healthy vision spontaneously acknowledged ‘taking vision for granted’ and how this played a role in their opinion of any distinction.

"I never realised how important is vision… until this moment. In general… I'm a very practical person. I just… everything has a reason for me. Vision, work… and all the activities… But then I'm listening [to other people's] experiences that makes me think… it's more than that, like a lot. A lot." 

31-year-old male with healthy vision.

This sentiment aligned with comments shared by two participants with ocular disease about the appreciation of vision.

"It seems like amongst the people with vision loss, the distinction isn't as important because it's one in the same… But… if you're discussing it with someone without vision loss, they may not appreciate how vision loss affects your life. They might not appreciate that there is joy in seeing the sunset, or there is joy in reading. They might just… take it for granted." 

37-year-old female with macular dystrophy.

### Current sources of visual enjoyment

Most participants were able to provide an example that was considered to be a current source of inherent visual enjoyment for them, such as seeing the natural environment, viewing art and photography, watching movies/TV and seeing a theatre show or live production. Examples shared that related more to visually facilitated enjoyment included exercising and playing sports, doing arts and crafts, travelling and driving and reading. Some participants specified how their vision also facilitated the joy of social interactions, rather than inherent visual enjoyment that came directly from ‘seeing someone[‘s] smiling face or seeing someone's face’ (47-year-old female with Stargardt disease).

"I like socialising. I belong to different groups and so on… you can go out and look at people and talk to people." 

76-year-old female with healthy vision.

"Looking at my grandchildren and seeing their development and interacting with them. I have to get fairly close up to see their expressions. But even at a distance you can get some visual enjoyment. My 13 year old granddaughter had a leading role in [a] production the other day and although I couldn't see her expression, you know… I could see the way she was performing… So, that sort of interaction with grandchildren." 

76-year-old male with geographic atrophy.

Several participants shared how their vision enabled them to work and see the output of their work, which brought their joy.

"I teach exercise classes so being able to still see movement and people is important to me because otherwise I couldn't do my job. So still having my peripheral vision, I can still do my work and do it well. I'm helping people regain movement or maintain their independence. So that's really enjoyable for me to be able to do that work with the vision that I have." 

47-year-old female with Stargardt disease.

### Importance of sources of visual enjoyment

In relation to their response to their current sources of visual enjoyment, most participants agreed that these were important to experience. Across groups, participants commonly reported that sources of visual enjoyment supported overall wellbeing, happiness and meaning in life.

"Well, the pleasure I get from say, going out for a walk… I explore what's around me… I'm intrigued by geology. I like to look at views. I like to look at plants, see what's around… see the evidence of the fireplace, trees and… the geology of the area … So it's actually seeing these things and the sort of satisfaction I get…10 out of 10. I mean, that's to me, that's essential… for life satisfaction, for meaning in life, being able to see these things. It's very important. 10 out of 10." 

70-year-old male with primary open angle glaucoma.

"The enjoyment for the landscape and the nature… it gives you peace and quiet and it's good for you. You can feel it… it's, it's that calmness and happiness, that deep, deeper thing."

70-year-old female with geographic atrophy.

For the people with healthy vision, a few participants commented on the importance of visual enjoyment to counter the stressors or monotony of their work.

"I spend a lot of time indoors… With star gazing… it's one of the opportunities I have… to actually be outside… I spend a lot of time in an office, so… it's a very nice break… like seeing life and technicolour sometimes when you've been staring at beige walls all day."

32-year-old male with healthy vision

Related to the importance of vision in facilitating enjoyable social interactions, participants across groups shared how vision helped them to feel more comfortable interacting with others (e.g., identifying social cues) and allowed them to engage in shared visual enjoyment (e.g., looking at a picture book with young children). Sources of visual enjoyment were important to these people because it enabled connection with others.

"When it comes to being able to see things and enjoy hobbies, it's hard not being able to talk about it with other people, especially when they ask, what have you watched lately? Have you seen this movie? Or there's references to pop culture and, because you haven't seen it, then you don't know what other people are talking about. So, it makes socialising uncomfortable sometimes."

37-year-old female with macular dystrophy.

### Changes to sources of visual enjoyment over time due to aging or vision loss

Most participants with healthy vision shared that their reported sources of visual enjoyment had changed over time due to evolving personal interests (e.g., more interested in the news, more appreciative of nature, having more free time) or external factors such as the COVID-19 pandemic or moving house.

"I realise that most of my life, I rushed. And I didn't stop and I didn't look around me… I had so many things: studying, working, bringing up kids, different countries. And I didn't have the impression that I appreciate life that I'm doing now… I'm retired. If I want to sit and look at a tree, I can. And I can look at every single [leaf] if I feel like it."

70-year-old female with healthy vision.

Some participants with healthy vision also commented on changes to their sources of visual enjoyment, presumably related to physiological age-related changes in vision with presbyopia and/or cataract development.

"Well… a lot of different things in terms of the practicality, because obviously my vision has changed, and I don't enjoy driving at nighttime. I do it because I have to, but it's not enjoyable… I [also] can't read the tablets. You know, if you have… something on the bottom of the tablet, you've got the impression you need a big magnifying glass."

70-year-old female with healthy vision.

Several participants with an ocular disease diagnosis highlighted a change in their sources of visual enjoyment, as well as the level of visual enjoyment they experienced, due to their vision loss. There were examples of a sense of frustration trying to engage in certain activities that had previously brought them visual enjoyment, alongside a fear of experiencing further decline in visual enjoyment as their vision loss progresses.

"When looking back on my photos… if I try to see them on my phone, I can't see them clearly enough and it has to be plugged into the big TV so that I can actually see the pictures that I've taken while we're away… I can do that now, but how long… I can remember when we were going across the Nullarbor and we went and saw all the whales congregating and that was just so amazing… All around Australia, it's just been amazing and it's… I'm afraid that I'm not going to see all of that." 

64-year-old female with Stargardt disease.

Other participants with vision loss due to ocular disease shared how they had adjusted. Adjustments were made so they could continue experiencing a particular source of visual enjoyment or to experience a different source of visual enjoyment, as well as adjusting expectations of how much visual enjoyment can be experienced.

"My son does graphics design. It's all football and basketball and… I used to be able to see his drawings with a lot of detail and now I can't quite. But he blows it up huge for me. So there's, there's still ways around things." 

47-year-old female with Stargardt disease.

"Because my vision has been deteriorating, I've noticed the change in my enjoyment of certain activities. So I think it would be different if… my whole life, my vision had been… stable… if it hadn't changed… So now I know… I've just adjusted… If I can't enjoy something the same way I have before, then I [find] something else that I can enjoy. But vision is like, it's like bending your elbow… You can bend your arm and then it can facilitate doing something else that you enjoy." 

37-year-old female with macular dystrophy.

"Your functional ability may be falling, but your enjoyment may also be falling… I mean… if [my partner and I are] both looking at a sunset, for example, I may still get some enjoyment from it, but I know I won't be getting quite the same enjoyment that [they] will be getting, because [their] vision is so much better… I like opera as well… but because you can't read the subtitles… you have to try to remember the plot. You sort of rebalance your proportion of enjoyment, I guess. You listen to the music more carefully."

76-year-old male with geographic atrophy.

### Role of eye care providers in addressing visual enjoyment

Some participants described their eye care providers addressing more broadly ‘what was important to me’ (80-year-old female with primary open angle glaucoma), but not ‘visual enjoyment’ per se. Most participants across both groups had minimal-to-no experience of their eye care providers addressing visual enjoyment, although some people questioned the relevance of their own eye care provider considering visual enjoyment.

"I don't know if it is really relevant, that [my eye care provider] knows what my enjoyment is. I think [they are] just doing everything [they] can to save my sight… I don't know if it's relevant to [them]."

65-year-old female with primary open angle glaucoma.

Most participants responded with a mix of perceptions as to whether they felt eye care providers should address visual enjoyment with their patients. Some of the possible benefits shared by those who were supportive related to enhanced quality of life and wellbeing and improved clinical decision-making.

"I think we probably all agree that it… that question has been missing… for most of us. And it seems to me that that's one aspect, no matter how busy the [eye care provider]… it would be a nice inclusion [for the] practitioners to be able to address that question… Surely… there's a huge need for just that little thing, to talk about enjoyment for you, because that's you. You're not a book, or a number or anything else, you're you. So, I feel it's critically important to address… that question. Maybe through teaching, maybe through university training. But it makes a difference to everybody at the table, so, therefore, the whole community out there would benefit from somebody who has [an] understanding of that question." 

80-year-old female with primary open angle glaucoma.

A few participants commented on the value of a health professional addressing visual enjoyment. For some people with ocular disease, the role may not necessarily be for the eye care professional who provides their functional assessments. For other participants, examples were given of how eye care professionals could include consideration of visual enjoyment as part of their testing.

"It's more of a social issue… rather than a medical, trying to save your eyesight… Maybe there needs to be yet another person in the office to give you counselling along those lines, rather than the doctor who's trying to operate upon an eye, [who] sort of does everything." 

80-year-old female with primary open angle glaucoma.

"[Another person] made a point earlier about adaptation and I think, you know, receiving advice about how to maximise enjoyment while experiencing a deterioration in your eyesight: any advice of that sort, I think… would be very helpful… Of course, people's preferences may differ, but if the professionals were able to say, ‘well, look, over time these things will happen. But here are some suggestions or people you could talk to that may… maximise your adaptation and maintain your enjoyment’… I think that's a very powerful point." 

76-year-old male with geographic atrophy.

"I think a good idea would be to include pictures or videos that bring enjoyment for most of the people to also measure how visual loss affects the feelings and mental health of patients… in addition to the common white chart with letters to read." 

33-year-old female with healthy vision.

For participants who felt that eye care providers should focus only on visual function, a few participants from each group commented on the constraints with appointment times if this were to be included and felt that addressing visual enjoyment is outside of the scope of eye care provision.

"They're not going to be my psychologist… I'm just here to get my eyes tested… I think the only thing… eye care [professionals] need to be concerned about is [my] capacity for driving."

76-year-old female with healthy vision.

### A positive perspective on vision loss

Although the negative aspects of vision loss were discussed by the participants, altered perception was not uniformly perceived as a negative compared with normally sighted observers, as highlighted by quotes from a participant with vision loss who also created painted artworks:

"… nobody sees the same thing the same way. So, it doesn't matter how your partner sees a sunset because she will never have seen it, the sunset, your way and vice versa."

"So, whatever we do, it's what it means to us, not anybody else's perception of what it should be like."

"And then we go out and we look at things and they say, “Oh, but you can't see them, you can't see that.” And I say, “Don't say that. I can see my way and you see your way.” So, it's forever teaching each other how to enjoy life and make the best of whatever you've got."

77-year-old female with geographic atrophy.

## DISCUSSION

This study represents the first attempt to try to separate enjoyment arising from our sense of vision (inherent visual enjoyment) from enjoyment facilitated by, or requiring, the ability to see (visually facilitated enjoyment), both in those with healthy vision and with vision loss. It was found that most people were able to think of situations or activities in which enjoyment was principally obtained from their visual sense. Most also felt that their sources of visual enjoyment were important to them. Despite this importance, personal sources of enjoyment were typically not considered when people visited their eye care providers.

For some activities that participants identified—such as enjoying seasonal colour changes of leaves or admiring the painted faces in Chinese opera—classifying these as inherent visual enjoyment was reasonably clear. However, for other activities, a distinction was less clear, with some aspect of enjoyment deriving from the sense of vision whereas other aspects of enjoyment deriving from the activity that is dependent upon vision. Examples raised in our focus groups included driving and playing golf—both of which could have enjoyable components falling under the heading of inherent visual enjoyment (e.g., enjoying the vista of the scenery whilst driving or on the golf course) but could also be deemed enjoyable activities without such a component. Due to time limitations, often it was not possible to disambiguate these when a participant volunteered this sort of activity. That many activities might contain a mix of enjoyment types may explain why some participants struggled to separate enjoyment arising directly from the visual sense from enjoyed activities that also involved vision.

In the present investigation, many participants reported a decrease in visual enjoyment because of vision loss. A recent study has highlighted that even moderate vision loss can impact on the ability to engage with and enjoy painted artworks.^13^ However, it should be noted that not all reflections on vision loss were negative, as noted in our results under ‘A positive perspective on vision loss’.

The focus groups also revealed that some participants had not previously considered the role vision had in providing enjoyment, much less whether vision facilitated their enjoyable activities or was an intrinsic source of enjoyment. It is likely that concepts of visual enjoyment would become more familiar to people—and so they might be able to provide more considered responses—should eye care providers not only investigate whether their patients' vision is sufficient to perform key functional tasks but also to provide enjoyment. Given this lack of previous consideration, these results may represent an underestimation of both the role of visual enjoyment and its importance in patients' lives and the potential importance of having visual enjoyment investigated by eye care providers. Despite this, most people felt that sources of visual enjoyment—whether intrinsic or visually facilitated—were important to them. This suggests there is scope for developing tools to quantify visual enjoyment specifically so that the impact of visual disease and the effectiveness of their treatments can be assessed more fully, given that existing survey and activities of daily living tools provide very little assessment of visual enjoyment.^2,4,16^

In this study, participants with vision loss and those with healthy vision were separated. Although matrix coding was used to compare groups, the analysis did not reveal any distinct differences. The findings indicate that both groups had similar thoughts regarding what constitutes sources of visual enjoyment, their importance and distinctness from visual function and whether eye care providers should consider visual enjoyment. Further studies with a larger sample size may consider exploring this further to determine whether more nuanced or subgroup-specific differences emerge that were not detectable in our current sample.

This study has several strengths. It included several causes (glaucoma, wet and dry age-related macular degeneration and inherited retinal degenerations) and types of vision loss (central and peripheral visual field). Although the median age of participants was around 70 years, it also included voices from those of working age, with approximately a third of the cohort being under 50 years of age (including three people with vision impairment). The sample size, which was deemed sufficient to tell a meaningful story,^15^ was also comparable to that used in other qualitative studies. Previous work found that an average of 12 to 13 interviews achieved data saturation when interviewing roughly homogenous groups.^21^ Approximately twice this number of participants was included, given the anticipated heterogeneity between those with and without vision loss. However, there are several limitations to this study. Participants were limited to those who could speak English and could attend in person in Melbourne. As with all qualitative methods, there is subjectivity in how responses were characterised. Time constraints limited the degree to which each individual's response could be explored, and so clarification on some responses was not always obtained. After the initial question regarding sources of visual enjoyment, responses to follow-up questions related to sources of visual enjoyment (e.g., changes with age of disease) may not have always related specifically to visual enjoyment, with some participants with vision loss changes speaking about general changes to their lifestyle.

In summary, people with and without vision loss and across a wide age range commonly felt sources of visual enjoyment were important but typically were not considered by eye care providers. Although not universally considered by the group to be the role of eye care providers, many participants felt that such consideration would be valuable in enhancing quality of life and clinical decision-making. Therefore, consideration of whether a person's vision is not only sufficient to perform functional tasks, but also to provide enjoyment, may provide a fuller assessment of the impact of vision loss.

## Supplementary Information

**Appendix S1** (PDF 71.6 KB)

**Appendix S2** (DOCX 59.2 KB)

**Table S1** (PDF 134 KB)

## Data Availability

The datasets analysed in Table 1 are available in the Figshare repository https://figshare.unimelb.edu.au/articles/dataset/Visual_Enjoyment_Workshop_Data/29232611.

## References

[1] GBD 2019 Blindness and Vision Impairment Collaborators. Trends in prevalence of blindness and distance and near vision impairment over 30 years: an analysis for the global burden of disease study. *Lancet Glob Health.* 2021;9:e130–e143. 33275950 10.1016/S2214-109X(20)30425-3 PMC7820390

[2] Haymes SA, Johnston AW, Heyes AD. The development of the Melbourne low-vision ADL index: a measure of vision disability. *Invest Ophthalmol Vis Sci.* 2001;42:1215–1225. 11328730

[3] Finger RP, McSweeney SC, Deverell L, O'Hare F, Bentley SA, Luu CD, et al. Developing an instrumental activities of daily living tool as part of the low vision assessment of daily activities protocol. *Invest Ophthalmol Vis Sci.* 2014;55:8458–8466. 25425306 10.1167/iovs.14-14732

[4] Mangione CM, Lee PP, Gutierrez PR, Spritzer K, Berry S, Hays RD. Development of the 25-item National Eye Institute Visual Function Questionnaire. *Arch Ophthalmol.* 2001;119:1050–1058. 11448327 10.1001/archopht.119.7.1050

[5] U.S. Food and Drug Administration. Principles for selecting, developing, modifying and adapting patient-reported outcome instruments for use in medical device evaluation: guidelines for industry and Food and Drug Administration Staff and other stakeholders. Silver Spring, Maryland, USA: U.S. Food and Drug Administration; 2022. p. 1–13.

[6] Massof RW, Ahmadian L, Grover LL, Deremeik JT, Goldstein JE, Rainey C, et al. The activity inventory: an adaptive visual function questionnaire. *Optom Vis Sci.* 2007;84:763–774. 17700339 10.1097/OPX.0b013e3181339efd PMC6742517

[7] Bruijning J, van Nispen R, Verstraten P, van Rens G. A Dutch ICF version of the activity inventory: results from focus groups with visually impaired persons and experts. *Ophthalmic Epidemiol.* 2010;17:366–377. 21090911 10.3109/09286586.2010.528133

[8] Massof RW, Hsu CT, Baker FH, Barnett GD, Park WL, Deremeik JT, et al. Visual disability variables. II: the difficulty of tasks for a sample of low-vision patients. *Arch Phys Med Rehabil.* 2005;86:954–967. 15895342 10.1016/j.apmr.2004.09.017

[9] Keeffe JE, Lam D, Cheung A, Dinh T, McCarty CA. Impact of vision impairment on functioning. *Aust N Z J Ophthalmol.* 1998;26:S16–S18. 9685012 10.1111/j.1442-9071.1998.tb01360.x

[10] Australian Bureau of Statistics. 4172.0 – Arts and culture in Australia: a statistical overview, 2008. 1st ed. Canberra: Australian Bureau of Statistics; 2008.

[11] Erickson-Davis, C, Korzybska H. What do blind people “see” with retinal prostheses? Observations and qualitative reports of epiretinal implant users. *PLoS One.* 2021;16:e0229189. 10.1371/journal.pone.0229189 33566851 10.1371/journal.pone.0229189 PMC7875418

[12] National Academies of Sciences, Engineering, and Medicine. The impact of vision loss. In: A Welp, RB Woodbury, MA McCoy, SM Teutsch, editors. Making eye health a population health imperative: vision for tomorrow. Washington (DC): The National Academies Press; 2016. p. 135–162. 27656731

[13] Cheng MJ, Rohan EMF, Rai BB, Sabeti F, Maddess T, Lane J. The experience of visual art for people living with mild-to-moderate vision loss. *Arts Health.* 2024;16:147–166. 37012640 10.1080/17533015.2023.2192741

[14] Tong A, Sainsbury P, Craig J. Consolidated criteria for reporting qualitative research (COREQ): a 32-item checklist for interviews and focus groups. *Int J Qual Health Care.* 2007;19:349–357. 17872937 10.1093/intqhc/mzm042

[15] Malterud K, Siersma VD, Guassora AD. Sample size in qualitative interview studies: guided by information power. *Qual Health Res.* 2016;26:1753–1760. 26613970 10.1177/1049732315617444

[16] Weih LM, Hassell JB, Keeffe J. Assessment of the impact of vision impairment. *Invest Ophthalmol Vis Sci.* 2002;43:927–935. 11923230

[17] Elo S, Kyngas H. The qualitative content analysis process. *J Adv Nurs.* 2008;62:107–115. 18352969 10.1111/j.1365-2648.2007.04569.x

[18] Sandelowski M, Voils CI, Knafl G. On quantitizing. *J Mixed Methods Res.* 2009;3:208–222. 10.1177/1558689809334210 PMC2768355 19865603

[19] Sandelowski M. Real qualitative researchers do not count: the use of numbers in qualitative research. *Res Nurs Health.* 2001;24:230–240. 11526621 10.1002/nur.1025

[20] Kegler MC, Raskind IG, Comeau DL, Griffith DM, Cooper HLF, Shelton RC. Study design and use of inquiry frameworks in qualitative research published in Health Education & Behavior. *Health Educ Behav.* 2019;46:24–31. 30227081 10.1177/1090198118795018 PMC6386610

[21] Hennink M, Kaiser BN. Sample sizes for saturation in qualitative research: a systematic review of empirical tests. *Soc Sci Med.* 2022;292:114523. 10.1016/j.socscimed.2021.114523 34785096 10.1016/j.socscimed.2021.114523

