# Supplementary material for: Seeing a sunset: Exploring the joy of vision, in healthy eyes and ocular disease
Source: Ophthalmic Physiol Opt. 2025 Sep 16;45(7):1703–14. doi: 10.1111/opo.70019 (PMC12682105; doi:10.1111/opo.70019)
Supplement: Supplementary file 1 — Appendix S1 (PDF 71.6 KB) [file 44402_2025_4507014_MOESM1_ESM.pdf]

Appendix 1: Focus Group Discussion Protocol

Thankyou & welcome statements

Introduction to the team of researchers

Distress protocol

“We hope that you find this workshop is a valuable opportunity to share your thoughts on what is an under-explored aspect of vision and vision loss, and that you are encouraged by the opportunity to help eye care providers and vision researchers better understand what people value from their vision, and how this may be impacted by vision loss.

We appreciate that some people may find that reflecting on what activities are a source of visual joy is distressing, particularly if they are no longer able to perform these activities. Please note that:

- you can choose to not answer any of the workshop questions
- you can choose to leave the workshop at any time
- all transcripts will be deidentified after the workshop, and comments cannot be traced back to a particular participant “

Study description

“Difficulty seeing – or vision loss - commonly occurs in many diseases and disorders that affect our eyes. Much work has been done on how best to quantify vision loss through various vision tests. Additionally, the impact of vision loss on the ability to perform various functional tasks has been well studied; for example, being able to read signs and labels,

navigating safely through streets, and being able to sort money. You completed a questionnaire prior to attending today that aimed to quantify some of these tasks.

However, less research has been done looking what are the things we do with our sight that are an inherent source of enjoyment, but that might not have a functional role. Examples might include

- looking at an artwork, or a sunset
- taking in the beauty of a streetscape or landscape, rather than simply being able to navigate through it
- the joy of seeing a loved-one's face, rather than simply being able to recognize it, or
- watching the face of a sleeping grandchild

Some of these tasks might be quite important to us, even though they don't serve any direct function other than providing us enjoyment or pleasure or joy. In this study we will be exploring what are sources of visual enjoyment, and how these may be influenced by vision loss."

Outline of Workshop Structure

- You will be working in one of two groups
    - Facilitator to ask initial questions
    - To encourage participation, and to ensure that all have an opportunity to speak, we will first seek answers by going around the group one at a time. Time permitting, we with then go around the group at least a further time so that people have the opportunity to comment further, based on what they have heard from the group.

    - We should have plenty of time for discussion, but please don’t be offended if facilitator does sometimes need to move the group on to a different question in the interests of time

- Outline of timetable (initial round of questions, lunch, concluding round of questions, debrief session)

- Reminder about session recording (allows subsequent transcription for analysis, with the recordings then being destroyed)
