# Supplementary material for: Seeing a sunset: Exploring the joy of vision, in healthy eyes and ocular disease
Source: Ophthalmic Physiol Opt. 2025 Sep 16;45(7):1703–14. doi: 10.1111/opo.70019 (PMC12682105; doi:10.1111/opo.70019)
Supplement: Supplementary file 2 — Appendix S2 (DOCX 59.2 KB) [file 44402_2025_4507014_MOESM2_ESM.docx]

**Appendix 2: Discussion Questions**

*(session prior to lunch)*

1. “I'd like you to take a minute or two to think about an activity or situation which you associate with visual enjoyment. In a moment we will go around the group to share what activity or situation we have each thought of. As we do, it is important that you stick with your original thought and do not change it in light of what you hear from others. It doesn't matter if your response is exactly the same, or completely different from others in the group. Do note that we will have the opportunity for a more general discussion about sources of visual enjoyment shortly. Again, please take a minute or two to think about an activity or situation which you associate with visual enjoyment, and then I'll ask each person that question.”
2. “As discussed at the in the introduction to this workshop, vision is commonly used to help us perform particular tasks, such as reading labels or navigating through a street. But vision can also be a source of enjoyment that may be unrelated to performing a particular task. For example, viewing a sunset or a piece of art. Do you think this distinction is important?”

*(session after lunch)*

1. “What are current sources of visual enjoyment for you and how important to you are these sources of visual enjoyment?”
2. *Different questions, based on group*
   1. *[Ocular disease group]* “Has the presence of visual vision loss influenced *previous* sources of visual enjoyment for you and have your sources of visual enjoyment changed?”
   2. *[Self-reported healthy vision group]* “Has what you consider to be a source of visual enjoyment changed as you have aged?”
3. “When you visit your eyecare provider, do you feel that sources of visual enjoyment for you are understood and considered when they provide advice? Is this important? If you don’t have an eye-care provider, consider this question from the perspective of how you think you would like your eye-care provider to act.”
