# Supplementary material for: Seeing a sunset: Exploring the joy of vision, in healthy eyes and ocular disease
Source: Ophthalmic Physiol Opt. 2025 Sep 16;45(7):1703–14. doi: 10.1111/opo.70019 (PMC12682105; doi:10.1111/opo.70019)
Supplement: Supplementary file 3 — Table S1 (PDF 134 KB) [file 44402_2025_4507014_MOESM3_ESM.pdf]

Online Supplementary Table. Description of codes and sub-codes, and illustrative quotes.

Code	Sub-codes	Participants with healthy vision	Participants with ocular disease diagnosis
1. Initial activity or situation associated with visual enjoyment	Inherent visual enjoyment	*"The first thing on top of my head was, I was looking outside of the window when I arrived here. There was the street and the colour of different leaves, and the blue sky today is really good. It's really beautiful today."* – 44-year-old female<br><br>*"For me, stargazing… I could spend hours looking up at the sky at night."* – 32-year-old male<br><br>*"I love nature, anything to do with nature, the gardens, the skies, the birds, the fish. So anything to do with colours, like autumn, the red, lovely colours. And sometimes in winter you see brown trees, but there is still a sculpture to it."* – 65-year-old female<br><br>*"Definitely all the big things, you know, see my kids or the dog chasing the ball… that's all fabulous. Go to the park and you've got this vista of people and dogs and kids… I love it."* – 76-year-old female<br><br>*"I love watching my grandchildren interact with each other and with my wife and I. It's just gorgeous."* – 73-year-old male	*"For me, I live in [name of location] and I love that beach view… When I come to the beach, it doesn't matter what the clouds are doing. Beach. Scenery."* – 70-year-old female with geographic atrophy<br><br>*"I'm a bushwalker, and… every Monday, I walk along the beach and I enjoy seeing the different seasons. It's windy, the grasses all blow. There's all different birds. This Monday, there were pelicans and seagulls, ocean gulls and swans, and other birds. I didn't know what they were, but I enjoy that every Monday, which I've enjoyed more and more as time goes on."* – 80-year-old female with glaucoma<br><br>*"Mine's fairly simple. It's seeing someone['s] smiling face or seeing someone's face."* – 47-year-old female with Stargardt disease<br><br>*"I love looking at the sea every day. Every day I'll look at the sea, which is always something interesting. Ships going in and out. Just love that."* – 74-year-old male with neovascular age-related macular degeneration
	Visually facilitated enjoyment	*"My enjoyment comes from travelling to places where the culture is different from what we have here."* – 81-year-old male<br><br>*"I love watching sport… a bit of football [where] you gotta… really focus. Yeah. So that's good."* – 76-year-old female<br><br>*"So, I play soccer. And it's a different type of enjoyment that you really get from the sports or even simple running… you get to enhance your ability to absorb more*	*"I think probably… reading. I spent most of my life, for work purposes and for enjoyment, reading. Particularly fond of history… So, the enjoyment from reading is, you know, the absorption of information, both for pleasure and previously for work purposes."* – 76-year-old male with geographic atrophy

		information from vision… it's good. It's a joy." – 40-year-old male	
2. Distinction between visual function and visual enjoyment	Conceptualization of the difference between vision for enjoyment and vision for function	*"I think there's two aspects here. There's the enjoyment, the aesthetics, if you like, which is what you see and what stimulates your imagination. On the other hand, there's the communication side, reading. It doesn't necessarily stimulate your aesthetic side, but it does stimulate your intellect. I think that there are two quite distinct applications [for vision]."* – 81-year-old male<br><br>*"Vision to me is different when at work and at home. So at work, it's more like a purpose. I've got standard operating instructions. Then after writing it, I present it to the student. And if I can see them doing the right thing, then I'm happy. Then when I'm at home, I'll go for walks with my husband. We enjoy the scenery, so it's vision for enjoyment."* – 65-year-old female	*"Definitely, because there are two distinct functions, you know, just the day-to-day achieving our aims. But then also there [are] these things everyone has described in the vision of seeing the sunset or the beach or the ships going around. And they're things that give you really good feelings."* – 70-year-old male with primary open angle glaucoma
	Acknowledgement that vision can have different roles but the concepts of vision for enjoyment and vision for function are intertwined	*"To my mind, there's overlap. You read something and it… the intellectual aspect is important, but then as your mind absorbs and changes, it becomes a more aesthetic… But reading a page of text doesn't necessarily stimulate the pictorial, the aesthetic side, but it does stimulate the intellect."* – 81-year-old male	*"I think you can draw some distinction. But I agree… that there's some overlap. So, for example, you may get enjoyment… from performing a function which requires vision. So, I'm trying to learn to cook, for example… But like the bushwalkers, I think that's where the distinction does apply… The vision limits how you walk —you know, whether you trip over things — but it also limits your ability to look and see things and enjoy, and I get that part of the enjoyment of bushwalking. So I think some distinction is useful, but I don't think I'd push it too far. There's a bit of overlap."* – 76-year-old male with geographic atrophy<br><br>*"Yes, it overlaps. Like, for example, I used to enjoy reading a lot. I can't read anymore. I used to enjoy the movie[s] and it's coming very much to an end. I persevere, but you know, only just... So this is also overlapping: the practicality, the function that I can do and the enjoyment you get out of it."* – 70-year-old female with geographic atrophy

	Acknowledgement that vision can have different roles but visual function is more important than visual enjoyment	*"If I lost my vision fully, I think the functional side of it would be more concerning to me than the enjoyment side… But again, you don't know, but that's how I think at this stage, if I lost functionality. you know — couldn't drive, hundreds of other things."* – 79-year-old male	*"Vision is ultra important for practical and functionality, because you use it to do everything… For me, it's all about just practical."* – 41-year-old male with cone-rod dystrophy
	Both visual function and visual enjoyment are important, and the distinction between the two concepts is important	*"100% sure it's important… I had a very bad accident, motorcycle accident… For that you learnt that vision as a function is very important, because you know, with the head injuries and other injuries… your minimal function is very important for your survival. But beyond that, after recovery you want to enjoy your life, so the vision to… acquire the enjoyment is very important as well. So when we talk about vision, it's not only about our eyes, but… the whole neural pathway from our eyes to our head."* – 40-year-old male<br><br>*"For me, I think the distinction, it's very important because I think… nowadays, people get more busy, right? Like for me as well, I have so many projects. And I think it's important also to give you time to have these moments of visual enjoyment… sometimes I need it. It's like, OK, I need to go to see the sunset somewhere, you know? And it gives me peace and happiness… Maybe sometimes when you work and you… do daily things. You're not… aware of… this need… But I think it's very important to have this visual enjoyment… because it… helps you also in your mental health and those things."* – 33-year-old female	*"It's just become clear [to me] that the value of vision from practicality and the fact that you get pleasure out of seeing things is quite an important distinction. Not everyone will recognise that necessarily because we're all just doing, living life, but it really is significant because there is so much satisfaction and joy that you get from the aesthetic experience… it is important to separate those two aspects of vision out."* – 70-year-old male with primary open angle glaucoma<br><br>*"Yes, I think it's important to distinguish because if we focus on only one, the importance of the other could be left behind... Especially now, when time is changing and people talking more about mental health. Mental health can be impacted by enjoyment of things so I think it's important to talk about both."* – 37-year-old female with macular dystrophy
	Distinction not perceived or not considered important	*"Even when you just use your vision as a tool to examine something, that still give[s] you… different feelings… No matter what, no matter what different purpose I use for my vision, I still got different feelings from it. So I, for myself, I can't separate."* – 44-year-old female	*"My first reaction was, for the research, OK, but otherwise what's the difference? I mean… it doesn't matter to me… the vision is going… so it makes no difference if I were to label it this name or that name."* – 70-year-old female with geographic atrophy<br><br>*"I think we should not make a distinction… Well, you got [to] think of aesthetic beauty and all that. And, of course, vision is very, very important. But if you think in terms of*

			a purely functional aspect of the eye… I think function can also become aesthetic, you know… because if you are not able to see things, then you become very frustrated, like crossing a street. And… you don't have that enjoyment of walking or doing things and activities… You might want to draw a line but… I don't think it's quite a significant line." – 68-year-old male with neovascular age-related macular degeneration<br><br>*"I'm looking at it from my own personal situation, which I think compared to everybody else here, I'm extremely fortunate… I've not experienced wondering what it's like to not have functional enjoyment vision because… I've still got both. But it's getting limited as I get older. But I still keep it as one huge important single item. They're both equally important. No distinction."* – 80-year-old female with primary open angle glaucoma
	Relevance of vision impairment to distinction	*"For me, the top thing on my head is if I can't see my kids play together, I can't see my kids' not-so-pretty drawings and the… Mother's Day cards they make for me, I will feel very sad. On the other hand, if I can still have some visual capability to see… to read the books or to read the signs, for example, I think my life is still OK but just the lost, the happy part of my life."* – 44-year-old female	*"So, vision of a beautiful scenery. Can [it] give a sense of awe to you?... It's a psychological effect. And actually it's quite a profound psychological effect because that sense of awe can undo some distress in people. So when you feel really good you just [relax]. It's like a reset to your being. So it's that ability to see and obtain awe from particular things, is a very valuable thing. And if people don't have the opportunity, well, that's a disadvantage. They've lost that ability to obtain that awe."* – 70-year-old male with primary open angle glaucoma
3. Current sources of visual enjoyment	Inherent visual enjoyment	*"I really like to see photos or videos of products that are handmade and see the details of the work. As well as pictures of symmetric shapes or "perfect" combination of pieces."* – 33-year-old female<br><br>*"After a hard day's work, you want to go out into the garden, have a look at the green plants. It's so visually relaxing. Or look at anything. My fish, I've got fish in a tank… So that again, visually enjoying."* – 65-year-old female	*"I can look at this window here and see a fantastic view. Now that, you know, that's the sort of thing that I think, wow… I'm fortunate that I can look out the window and see that. The other thing that's really important to me is where I live… which at the moment has got a huge number of deciduous trees, and because we've had a mild autumn, these trees are just marvelous. So, for me to be able to walk my golden retriever dog and kick my way through these mountains of leaves is absolute, pure pure joy."* – 80-year-old female with primary open angle glaucoma

		"I can't add too much more to what people have said about the aesthetic side of visual observation. It's important to me that I like going to the Botanic Gardens and beautiful, restful, serene environment." – 81-year-old male<br><br>*"Another kind of enjoyment is from my house. The reason we chose our current house is that we have a large open space in front of [the] house, and… we have a large window there… Every time we want to relax or calm ourselves down, we just sit in front, look at the open space."* – 40-year-old male	*"I do love looking at beautiful paintings and artwork and all the beautiful books that come through the library. So all those special things that galleries [offer]… I really enjoy looking at… Because I'm not a creative person. I'm just in awe of people. And we have all sorts of different groups in the library, you know, doing crochet, knitting, lace making. I'm just always in awe of what they do…Anyone who can do anything like that, I think it's amazing. So I do get a lot of enjoyment out of seeing the amazing things that other people create."* – 65-year-old female with primary open angle glaucoma<br><br>*"Current sources of visual enjoyment… I guess I would get a lot of enjoyment being outdoors so we enjoy camping and being in nature. I walk the dog all the time and just seeing green is just happy. Just makes you feel great."* – 47-year-old female with Stargardt disease<br><br>*"We live right opposite a park. I walk out the front gate and I'm in a park, there's rainbow lorikeets and cockatoos and all sorts of things there. And then I walk through a building and I'm there on the beach, which once again, it's terrific to look at."* – 74-year-old male with neovascular age-related macular degeneration
	Visually facilitated enjoyment	*"I read a lot. For me, vision and enjoyment: it's completely together, because if I couldn't read, I would be really unhappy."* – 70-year-old female<br><br>*"Especially coming [from] my field, the power of visualization plays an important role… Being a student, the enjoyment is seeing your computer screen getting your results, colorful graphs."* – 26-year-old male<br><br>*"My visual enjoyment is being able to see the big world. Being able to get into that space. So that… could mean just going into the city or going down to the beach or being in the gym or being anywhere really… And having that capacity to get there as well. So it's also the visit,*	*"I do Wordle and Quordle every day… I have a large iPad, and you can blow up the script. I have to sort of navigate backwards and forwards, but that sort of visual enjoyment of being able to do those puzzles; yeah, I enjoy that."* – 76-year-old male with geographic atrophy

		the physical physicality of getting to those spaces and being able to enjoy what I'm seeing and to feel it. It doesn't matter what it is, really doesn't matter what I'm looking at… So it's vision… If I couldn't see or I couldn't get there because they go hand in hand… I can't imagine one without the other." – 76-year-old female	
	Importance of current sources of visual enjoyment	*"I work in IT, so I'm most of the time on the computer. It's very, very important for me to… give my eyes some rest and really make a time to enjoy… something beautiful in the nature… Even going to the balcony in the sunset or things like that, or when it's raining or seeing the cockatoos, all the animals, and the bats that are flying very close… I really, really enjoy that. And I think it's very important for me as well."* – 33-year-old female<br><br>*"Sometimes we get… very complex tasks [at work]. And it's interesting… I cannot think when I am just look[ing] at a screen, and in those moments I have to go outside and check the view, and that clears my mind… That gives me peace… to think clearer, and to start again. So that makes me very happy… [it] refreshes my everything for that moment. And then I can go back and think and start again… that's my lifeline."* – 31-year-old male<br><br>*"Recently, I enjoyed reading books with my daughter because she's five years old. And in it, you know, not only the words… it's actually words with pictures. So, we discuss a lot besides… words. Details of the pictures, and both of us have a different aspect, perception of… the book. So we really enjoyed [sharing it with] each other."* – 40-year-old male	*"Well, the pleasure I get from say, going out for a walk… I explore what's around me… I'm intrigued by geology. I like to look at views. I like to look at plants, see what's around… see the evidence of the fireplace, trees and… the geology of the area … So it's actually seeing these things and the sort of satisfaction I get...10 out of 10. I mean, that's to me, that's essential… for life satisfaction, for meaning in life, being able to see these things. It's very important. 10 out of 10."* – 70-year-old male with primary open angle glaucoma
4. Changes to sources of visual enjoyment	Changes to visual enjoyment with aging or vision loss	*"My reading capabilities seem to be decaying quite noticeably, and I can't read small print. I haven't tried using a magnifying glass but reading a paragraph in the newspaper is a challenge and I find my eyes are starting to water towards the end of the paragraph, and I have to give up. I feel that's a great loss because reading text material is so rewarding. And there are novels that I'd*	*"I used to enjoy journal writing. I kept a paper journal. Fancy paper, fountain pens. Trying to teach myself to write with my alternate hand, so I could get really good at my handwriting. But I've stopped, 'cause trying to read my writing has gotten really hard. Yeah, I just don't enjoy it now; it's more of a frustration, trying to journal."* – 37-year-old female with macular dystrophy

		like to read, but I know I won't get through a chapter and it's a real challenge." – 81-year-old male<br><br>*"I'm intrigued by the colors of the leaves and the sounds of the ducks… and the beauty of their plumage, and it's like that didn't seem to be there as much before … I guess that's partly because, as you acquire knowledge, you start to abstract things more, you start to see patterns and symbols and… stories being told, and architectural design elements, and being able to connect with culture at that level… I notice myself doing that more, being distracted towards those sorts of things: the simple things that are just there in your environment."* – 32-year old male	*"One of my hobbies always was to… look at people's faces… but I have no face recognition whatsoever. Even my son, I walk past in the street unless he comes up… I'm a movie buff. So that is coming to the point where I can't recognise my favourite actors anymore, you know, like coming to an end and that is a bit of a hard thing."* – 70-year-old female with geographic atrophy<br><br>*"The movies… I still go a tiny bit, but I sit in the first row now, so I'm not with my husband or the friends. I'm separated, and I feel very sad about that until somebody sat close to me in the first row. So, I asked that person, are you vision impaired? And they said, No, I like to be part of the atmosphere. So, as a way of coping with the sadness, I always tell myself, oh, no! You want to be part of the movie. So, I've turned it around a bit. But there's still that initial sadness about not sitting with the others."* – 77-year-old female with geographic atrophy
	Adapting to changes in visual enjoyment due to vision loss		*"Painting. I paint… much more than I used to. Ever since I've lost my vision… I find a different way of painting: I paint with my fingers instead of painting with [a] brush. That's my great joy, and very happy with organizing exhibition[s] and selling, and so it's been a great source of joy, pride."* – 77-year-old female with geographic atrophy<br><br>*"In terms of visual enjoyment, the environment has never been [a problem] because it's so huge. Whether you can see tiny detail or the big things, they're still amazing. So the environment for me is still as amazing as it always was, even if I've lost a bit of [vision]... We were at the beach and someone said, oh, there's dolphins jumping over there'. And I'm like, I can't see the dolphins, but it still looks beautiful'."* – 77-year-old female with Stargardt disease
5. Role of eyecare providers	Experience of visual enjoyment being addressed	*"It's the last thing on their mind… It doesn't bother me much. But no, it's never asked. No, they don't ask. Asking what book you're reading or… what TV show did*	*"When [my eyecare provider] told me that I had to do less reading, they didn't provide any other options, or how I could make it easier, or any alternative. I ended up*

		you [watch]? There's no conversation. There's no chat. Absolutely no chat." – 76-year-old female<br><br>*"I think they also assume that if you can read good, you're gonna see the rest of it good… But that's why, maybe… they don't [address visual enjoyment]."* – 33-year-old female<br><br>*"I don't think we consider this is as part of their job. So we just think, OK, get your eye check and then… your eyes are healthy. That's it… I'm not expecting them to do that."* – 44-year-old female	*trying to take [their] advice, but in the end, I just had to ignore their advice and continue doing what I was doing, no matter the consequences… We didn't talk about what I can do or how I can enjoy my life… And that's really depressing… I was really sad for a while, but I feel like I'm alone trying to adjust to this and it feel[s] pretty helpless, like I'm just kind of waiting for someone to tell me, this is what you can do… these are the tools that you can use… Yeah, the, the information isn't easily available, I don't really know where to look."* – 37-year-old female with macular dystrophy
	Preference for eyecare providers to focus on visual function rather than visual enjoyment	*"I feel like eyecare providers focus on the function, not on the enjoyment. And if your visual function is best, if they are able to correct your visual function, then I feel your enjoyment comes, follows it. If you are facing some difficulties…in viewing… some things, or not able to read, that comes as a function. And, obviously, to distinguish colours; that is, also, I feel it is a function. So, [from] my perspective, I value the eyecare providers to provide me the basic functions."* – 26-year-old male	*"I'm a little bit more cynical there, I would say. If I go to an [eyecare provider], I don't need the add on… I don't know, I don't need to tell them what I did yesterday or whatever. Like I go to an [eyecare provider] to have that checked and then I have my art therapist to do my other things. That's how I see it."* – 70-year-old female with geographic atrophy<br><br>*"[My eyecare provider is] more the practical engineering type… making sure [they get] everything right… I wouldn't say [they think] about the aesthetics. I mean, our conversations are more about practicalities of the vision rather than, oh what else, you know?... I think making sure that the system is functional and keeps its function [is their role] and that's what matters because… the aesthetics is up to me."* – 70-year-old male with primary open angle glaucoma
	Preference for, and perceived value of, addressing visual enjoyment	*"I think it is important… [I was] playing golf with a friend, and he does wear glasses for very minor refractive error, he has [the] same issue as me. And he suggested that I try his glasses just to help me see the ball… All of a sudden I wasn't just looking at the ball. I was noticing other aspects of the fairways that I wasn't able to really pay attention to as much. I felt that one question he asked, and that one suggestion he made… was worth the time invested, because the two visits I'd made [to the eyecare provider], I was just told that you've got a very*	*"I guess it would be useful if the [eyecare provider] did in fact inquire about what's going on. Because that would give more impetus for them to understand what's going on."* – 70-year-old male with primary open angle glaucoma<br><br>*"Having…a rehab background, I guess, when I deal with people physically, we're always looking at improving quality of life. So it would make sense to me that from a visual perspective that you also focus on the person's*

		minor refractive [error]. To correct that, it's up to you whether you want to get glasses or not. It's not that much of an issue. So, it's… up to me… I'm not a professional golfer, but I think it would be a nice feature to have this part of consults… there'd be a myriad of examples… that could improve someone's enjoyment of things." – 32-year-old male	*quality of life, whether it can change the outcome. But understanding what's important to that person I think is important, whether it happens in that appointment or whether it happens from other research or investigating other things. I think it's important to look at the person as a whole and treat them as a whole, not just some eyes."* – 47-year-old female with Stargardt disease<br><br>*"I had a bad experience… before I was diagnosed with my condition… I went to an [eyecare provider] looking for help… We didn't talk about enjoyment, and we only talked about the function of the eyes and what I could see… I think if… [they] had assisted me back then I probably would have gotten the help sooner than I needed."* – 37-year-old female with macular dystrophy
	Ways that health professionals could address visual enjoyment	*"I think a good idea would be to include pictures or videos that bring enjoyment for most of the people to also measure how visual loss affects the feelings and mental health of patients… in addition to the common white chart with letters to read."* – 33-year-old female	*"[Another person] made a point earlier about adaptation, and I think, you know, receiving advice about how to maximize enjoyment while experiencing a deterioration in your eyesight: any advice of that sort, I think… would be very helpful… Of course, people's preferences may differ, but if the professionals were able to say, well, look, over time these things will happen. But here are some suggestions or people you could talk to that may… maximize your adaptation and maintain your enjoyment'... I think that's a very powerful point."* – 76-year-old male with geographic atrophy
